# Bacterial community composition and diversity of two different forms of an organic residue of bioenergy crop

**DOI:** 10.7717/peerj.6768

**Published:** 2019-04-18

**Authors:** Matheus A.P. Cipriano, Afnan K.A. Suleiman, Adriana P.D. da Silveira, Janaína B. do Carmo, Eiko E. Kuramae

**Affiliations:** 1 Instituto Agronômico de Campinas (IAC), Campinas, Brazil; 2 Department of Microbial Ecology, Netherlands Institute of Ecology (NIOO-KNAW), Wageningen, Netherlands; 3 Universidade Federal de São Carlos, Sorocaba, Brazil

**Keywords:** Bacteria, 16S rRNA, Sugarcane, Vinasse, Diversity, Bacteria isolation, Biochemical characterization, Nitrogen cycle genes

## Abstract

The use of residue of sugarcane ethanol industry named vinasse in fertirrigation is an established and widespread practice in Brazil. Both non-concentrated vinasse (NCV) and concentrated vinasse (CV) are used in fertirrigation, particularly to replace the potassium fertilizer. Although studies on the chemical and organic composition of vinasse and their impact on nitrous oxide emissions when applied in soil have been carried out, no studies have evaluated the microbial community composition and diversity in different forms of vinasse. We assessed the bacterial community composition of NCV and CV by non-culturable and culturable approaches. The non-culturable bacterial community was assessed by next generation sequencing of the 16S rRNA gene and culturable community by isolation of bacterial strains and molecular and biochemical characterization. Additionally, we assessed in the bacterial strains the presence of genes of nitrogen cycle nitrification and denitrification pathways. The microbial community based on *16S rRNA* sequences of NCV was overrepresented by Bacilli and Negativicutes while CV was mainly represented by Bacilli class. The isolated strains from the two types of vinasse belong to class Bacilli, similar to *Lysinibacillus*, encode for *nirK* gene related to denitrification pathway. This study highlights the bacterial microbial composition particularly in CV what residue is currently recycled and recommended as a sustainable practice in sugarcane cultivation in the tropics.

## Introduction

Sugarcane is a bioenergy sustainable crop used to bioethanol production and is considered a sustainable biofuel (De Souza et al., 2014), especially because it can reduce the greenhouse gas (GHG) emissions when compared to fossil fuel (Boddey et al., 2008; Galdos et al., 2010). Brazil is one of the largest producers of sugarcane with a cultivated area of 10 million hectares and ethanol production of 11–28 million m^3^ (UNICA (União da Insdústria de Cana-de-açúcar), 2014; CONAB, 2017). However, some management practices may misbalance this sustainable strategy as ethanol production generates many agro and industry-residues such as sugarcane-straw, bagasse, molasses, filter cake and huge volumes of the organic pollutants vinasse (one L ethanol: 10–15 L vinasse) (Prado, Caione & Campos, 2013).

The sugarcane residue recycling practice follows the legal rules of the Brazilian environmental regulations (CETESB, 2014). The residue vinasse generated during the ethanol production is disposed in soil as organic fertilizer. Vinasse is rich in nutrients, especially in potassium (∼3,000 mg L^−1^) and has high organic loads (biochemical oxygen demand and chemical oxygen demand) and pH of 4–5 (Fuess & Garcia, 2014; De Oliveira et al., 2015). Due to vinasse chemical composition, it is a valuable organic residue to be recycled to increase soil quality and plant productivity. Besides the alternative of vinasse application as organic fertilizer in sugarcane plantations, vinasse is concentrated by evaporation without losses of nutrients and organic matter. In addition, the high water content in vinasse may cause soil and groundwater contamination. Thus, nowadays both non-concentrated vinasse (NCV) and concentrated vinasse (CV) are widely used in fertirrigation, particularly to replace the potassium fertilizer.

Despite the advantages of vinasse application on the field, vinasse fertilization can increase GHGs emissions mainly nitrous oxide (N_2_O), which emission factor can reach up to 3% of *N* applied in soil (Pitombo et al., 2016; Siqueira Neto et al., 2016; Lourenço et al., 2018a, 2018b). Vinasse fertilization, as organic residue and a source of nutrients, alters the soil bacterial community composition and function of different microbial groups (Suleiman et al., 2018), which can affect the key biogeochemical processes, in particular nitrification and denitrification. In recent studies we have identified (Pitombo et al., 2016; Cassman et al., 2018) that bacterial members belonging to Firmicutes phylum present in vinasse are potential denitrifiers contributing to N_2_O emissions.

While for many agriculture and industrial residues (e.g., municipal wastewater, swine manure) literature on microorganisms present in the organic residues is available (Suleiman et al., 2016), the knowledge of vinasse microbes is restricted. Ethanol industry does not occur in sterile conditions and the material originated from sugarcane-ethanol production include mainly different genus of Firmicutes phyla and *Acetobacter* (Alphaproteobacteria) associated with this industrial process (Costa et al., 2015; Brexó & Sant’Ana, 2017). Furthermore, the microbial communities in different batches of vinasses are known of having low alpha diversity with core genus of *Lactobacillus* and with the presence of potential genes for denitrification but not nitrification in the vinasse metagenomes (Cassman et al., 2018; Lourenço et al., 2018c).

Current researches revealed that fertirrigation with CV and NCV impacts the N_2_O emissions (Pitombo et al., 2016; Cassman et al., 2018; Lourenço et al., 2018a). The knowledge about vinasse microbiome can decipher important ecological and environmental issues as a potential invasion of these microbes in the resident soil microbial community, especially CV for which there is no information about the bacterial community diversity. We hypothesize that (1)NCV and CV harbor different bacterial communities and (2) both vinasses isolated microbes have potential genes related to denitrification process. Therefore, this study aimed to evaluate the (i) bacterial community present in NCV and CV by next generation sequencing of 16S rRNA gene amplicon, (ii) isolation of bacteria from both vinasse types and (iii) molecular and biochemical characterization of the strains as potential denitrifier sources.

## Material and methods

### Vinasse chemical composition, total DNA extraction and 16S rRNA gene amplification

Four replicates of non-concentrated vinasse (NCV1, NCV2, NCV3, NCV4) and four replicates of concentrated vinasse (CV1, CV2, CV3, CV4) were obtained from different batches of a single sugarcane mill localized in Piracicaba, Brazil (22°43′30″S, 47°38′51″W) by Cassman et al. (2018), which chemical composition of NCV and CV are shown in Table S1. For total DNA extraction, a total of 90 mL from each replicate of concentrated and NCVs were harvested and centrifuged at 2,000×*g* for 5 min. A 250 mg pellet of each replicate was used for total DNA extraction using kit PowerSoil kit (MoBio Laboratories, Inc., Carlsbad, CA, USA) according to manufacture. The DNA quantity and quality were determined by spectrophotometer NanoDrop 1000 (Thermo Scientific, Waltham, MA, USA).

The microbial community was determined based on V4 region of the 16S rRNA gene sequencing. The 16S rRNA was amplified by archaeal/bacterial primers 515F (5′-GTGCCAGCMGCCGCGGTAA-3′) and 806R (5′-GGACTACVSGGGTATCTAAT-3′). The 25 µL 25 PCR contained five µL of FastStart High Fidelity taq Enzyme, 10× FastStart High Fidelity buffer containing 18 mM MgCl2 (Roche Diagnostics Ltd., Burgess Hill, UK), 0.2 mM of each dNTP (Promega UK Ltd., de Southampton, UK) and 0.1M of each primer. Each PCR had one µL DNA of each sample. The PCR conditions were 95 °C for 5 mi., followed 35 cycles at 95 °C for 30 s, 50 °C for 30 s and 72 °C for 1 min, with a final extension at 72 °C for 10 min. The PCRs were in triplicates and amplified in a termocycler C1000 (Bio-Rad, Hemel Hempstead, UK). The PCR products were visualized on 1.5% agarose gel in TBE buffer. The PCRs were purified by QIAquick kit (Qiagen, Hilden, Germany), quantified with Quant-iT Broad-Range DNA Assay Kit (Invitrogen, Carlsbad, CA, USA) in conjunction with the BioTek Synergy HT microplate reader and combined in equimolar ratios. The *16S rRNA* amplicons were sequenced using Ion TorrentTM semiconductor technology chemistry for unidirectional sequencing of the amplicon libraries. Barcoded primers were used to multiplex the amplicon pools in order to be sequenced together and separated afterward. Template preparation was performed using Ion OneTouch 2 System and Ion PGM Template OT2 400 Kit, and sequencing using Ion PGM Sequencing 400 on Ion PGM System using Ion 318 Chip v2.

The sequences were analyzed by Mothur version 1.33.2 (Schloss, 2010) combined with Snakemaker (Koster & Rahmann, 2012). First, multiplexed reads were filtered for quality and assigned to samples by matching to barcode sequences. Second, the reads were trimmed including one mismatch to the barcode and two mismatches to the primer, eight maximum homopolymer, minimum length of 250 bp, maximum length of 290 and quality score >25. Then, the sequences were aligned using the Silva reference database (Quast et al., 2013) template, pre-clustered to eliminate sequences outside of desired range alignment and potentially chimeric sequences were removed. Sequences were classified using Silva rRNA database (release SSU_Ref_119) with a confidence threshold of 80% and the sequences classified as chloroplasts and mitochondria were removed. The data were then analyzed in R (R Development Core Team, 2005) using Phyloseq package (McMurdie & Holmes, 2013). The sequences are available at the European Nucleotide Archive (https://www.ebi.ac.uk/ena/) under the study accession no. PRJEB30243. Samples were then, rarefied to determine the alpha diversity and principal coordinate analysis (PCoA) based on beta diversity (Supplemental Methods). Homogeneity of multivariate dispersion was tested by PERMDISP (Anderson, 2006) to determine if the community in different vinasses was shaped by a stochastic or a deterministic processes and the analysis was performed in calypso software (Zakrzewski et al., 2017).

### Bacteria isolation, molecular and biochemical characterization

A volume of 100 µL of each replicate of NCV and CV were plated on MRS (Man, Rogos and Sharpe, Difco®) medium and incubated at 37 °C for 48 h. The pure colonies were characterized by Gram staining test. The presence of different enzymes and different organic compounds as carbon sources of the isolated bacteria were determined by API 20 NE test (BioMérieux AS, Lyon, França) according to manufacture.

**Figure 1:**
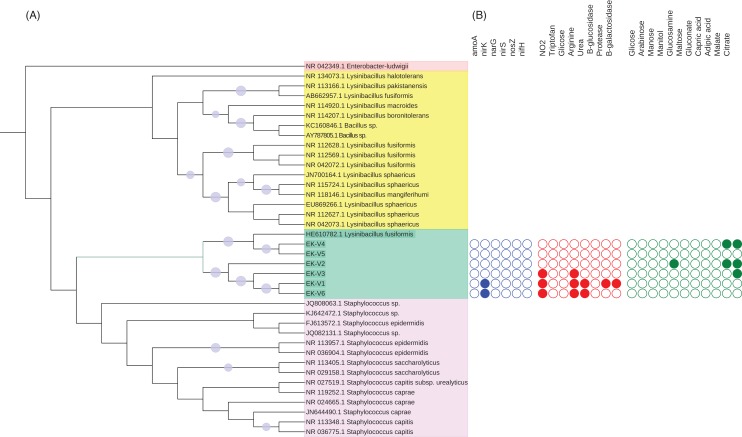
(A) Phylogenetic tree based on the alignment of 16S rRNA gene from vinasse strains and (B) representation of *N* cycle genes and biochemical characterization of substrates metabolized by the strains.

The DNA of each isolated bacteria was extracted using PowerSoil kit (MoBio laboratories, Inc., Carlsbad, CA, USA) for molecular identification by sequencing the 16S rRNA gene. The 16S rRNA gene was amplified with 27f (5′AGAGTTTGATCMTGGCTCAG3′) and 1100r (5′AGGGTTGGGGTGGTTG3′) primers using the PCR conditions described in Lane et al. (1985). The PCR products were purified by Gel Band Purification Kit (GE Healthcare, Chicago, IL, USA) and sequenced (Macrogen Inc., Seoul, Korea). The strains 16S rRNA sequences were blast against Genbank (http://www.ncbi.nlm.gov/GenBank) sequences. The 16S rRNA gene sequences (>85% identity) were downloaded from NCBI and aligned with the six strains 16S rRNA gene sequences using ClustalW in MEGA7 (Kumar, Stecher & Tamura, 2016). All the sequences were edited and aligned. A neighbor-joining tree (Saitou & Nei, 1987) was created to examine the phylogenetic relationships between the six strains and the sequences from NCBI using as outgroup an *Enterobacter ludwigii* sequence as show in Fig. 1. Distances were computed using the Maximum Composite Likelihood method and a bootstrap test with 1,000 replicates was conducted (Felsenstein, 1985). We used the Interactive Tree of Life (Letunic & Bork, 2016) to visualize the tree.

The potential contribution of the isolated strains on nitrogen cycle was determined by amplification of *amoA*, *nifH*, *narG*, *nirS*, *nirK* and *nosZ*. The primers and PCR conditions of each gene (Rotthauwe et al., 1997; Braker et al., 1998; Poly et al., 2001; Bru et al., 2007; Throback et al., 2004; Henry et al., 2006) are listed in Table S2.

## Results

### Vinasse microbial diversity and core microbiome

**Figure 2:**
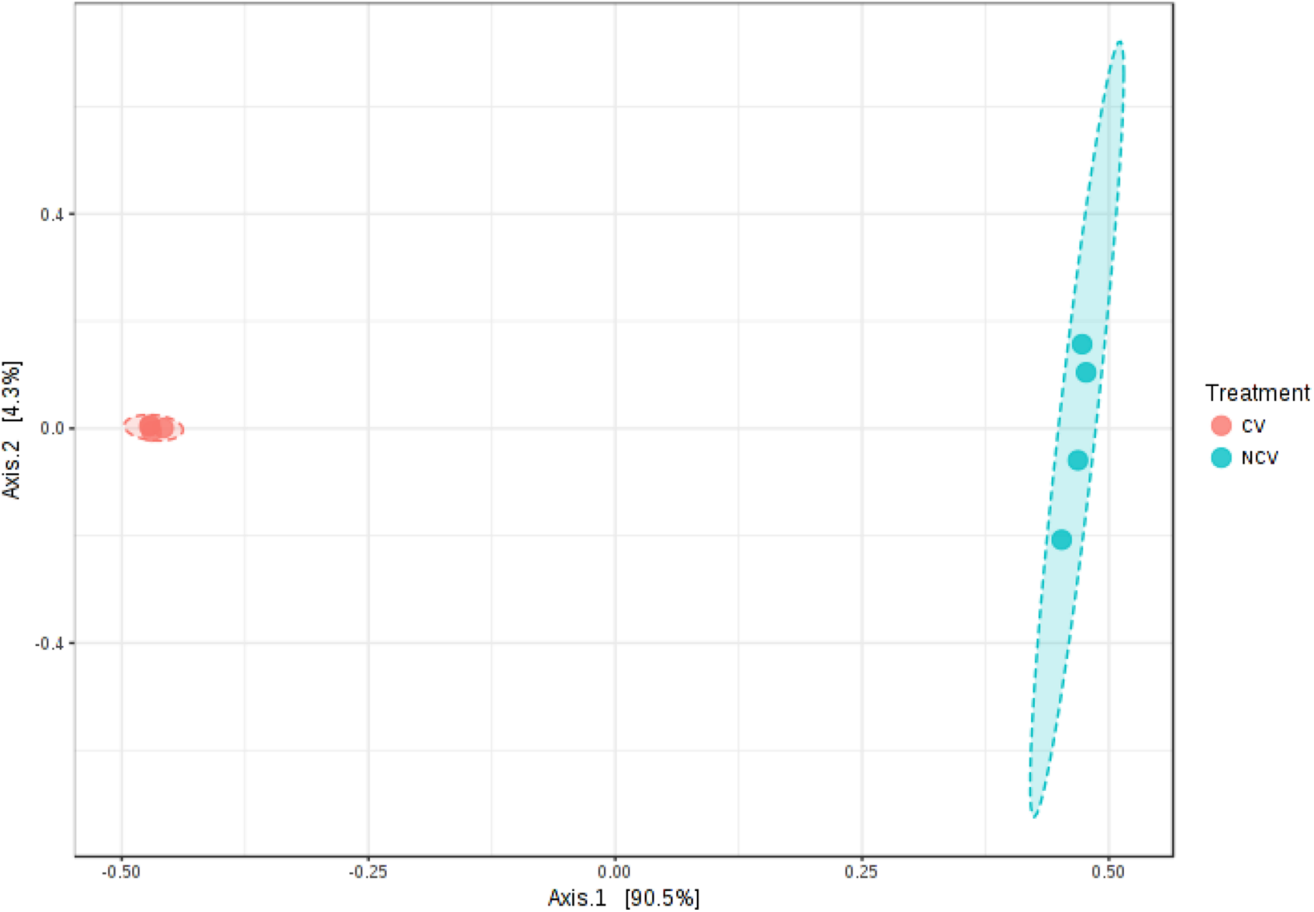
Principal coordinate analysis (PCoA) of bacterial communities based on the OTUs of the 16S rRNA gene sequencing from non-concentrated vinasse (NCV) and concentrated vinasse (CV). Bar charts of most abundant phyla, classes and genera of non-concentrated vinasse (NCV) concentrated vinasse (CV).

A total of 49,595 *16S rRNA* sequences were obtained for eight vinasse samples (four CV and four NCV) with average of 6,199, minimum of 3,688 and maximum of 9,496 sequences. Because differences in α- and β-diversities between NCV and CV can arise from differences in similarity, differences in dispersion or both, a separate test of dispersion using PERMDISP was used to detect the nature of such differences. The PERMDISP analysis revealed no significant difference in dispersion, that is, homogeneity of variance, suggesting that differences in alpha- and beta-diversities were largely driven by dissimilarity rather than dispersion (Fig. S1, *p* > 0.05). The alpha-diversity was higher in NCV than in the CV based on Shannon’s and Simpson’s indexes, Chao and for observed OTUs, despite being non-significant only for richness calculations (Fig. S2, *p* > 0.05). PCoA plots also showed that microbial communities grouped into two clusters (Fig. 2, Permanova *R* = 0.9, *p* < 0.05). The first two principal coordinates, PC1 and PC2, explained 94% and 2% of the data variation, respectively, clearly separating the communities of NCV and CV.

**Figure 3:**
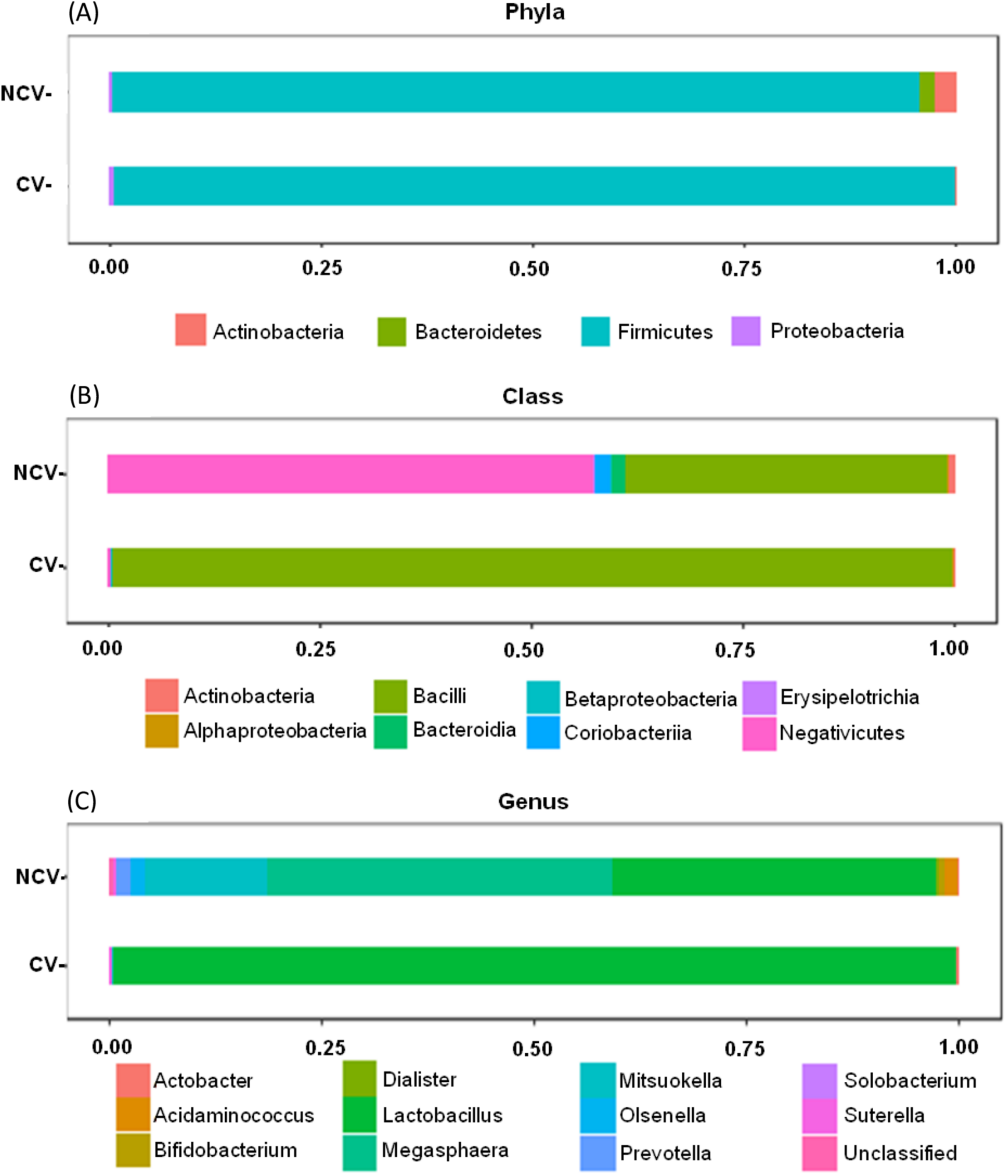
Bar charts of most abundant (A) phyla, (B) classes and (C) genera of non-concentrated vinasse (NCV) concentrated vinasse (CV).

Both NCV and CV showed dominance of phylum Firmicutes with 95% and 99%, respectively (Fig. 3A). However, at class taxonomic level, the distribution of the bacterial groups was different in NCV and CV (Fig. 3B). In NCV, the most abundant class was Negativicutes (50%), followed by Bacilli (38%) while in CV Bacilli corresponded to almost 100% of the total relative abundance. At genus level, NCV was represented by *Megasphaera* (43%), followed by *Lactobacillus* (40%) and *Mitsuokella* (18%) while CV was overrepresented by mainly *Lactobacillus* (Fig. 3C).

### Bacteria isolation from vinasses

Five strains (EK-V1, EK-V2, EK-V3, EK-V4 and EK-V5) were obtained from NCV and one strain (EK-V6) from CV. The phylogenetic tree based on 16S rRNA complete gene sequences of the strains showed two main distinct clusters (Fig. 1A). In cluster 1 are EK-V5 and EK-V4 strains that grouped with *Lysinibacillus fusiformes* and in cluster 2 are EK-V2, EK-V3, EK-V1 and EK-V6 strains. The PCR results of the *N* cycle genes of the six strains showed amplification only for *nirK* of strains EK-V1 and EK-V6 (Fig. 1B).

The Gram staining test showed that all strains are Gram positive. By API 20 NE test, the strains EK-V1, EK-V3 and EK-V6 are capable to reduce nitrate to nitrite and hydrolyze arginine; strains EK-V1 and EK-V6 are capable to metabolize urea; protease and B-galactosidase are metabolized only by EK-V1 strain (Fig. 1B). Regarding to organic compost assimilation only strain EK-V2 showed activity to glucosamine, while the same strain EK-V2 and EK-V4 were able to assimilate malate; the strains EK-V2, EK-V3 and EK-V4 were capable to assimilate citrate.

## Discussion

Organic fertilizers are usually complementary to inorganic fertilizers supplying the majority of plant nutrients needs. Thus, organic fertilizers are used in agriculture to improve the soil structure and stability in addition to enhancing the yield and, in particular, the quality of plants (Chang et al., 2010; Marzouk & Kassem, 2011). Vinasse organic fertilizer, for example, meets the specifications for organic farming with a high fertilizing value; it represents a considerable source of potassium but it also provides significant levels of phosphorus and nitrogen taken up by sugarcane plant (Christofoletti et al., 2013). Because of the large volumes of vinasse produced, concentration of vinasse by evaporation is an alternative to reduce the volume and avoid long transportation and soil compaction in the sugarcane fields (Christofoletti et al., 2013; De Carvalho et al., 2011). Our results showed that the process of vinasse concentration decreases bacterial diversity and change the microbial structure but still harbors bacteria with potential contribution to N_2_O emissions, based on the isolation of the bacteria strain EK-V6, which possess *nirK* (Fig. 1B). The strain EK-V1, isolated from NCV also possesses *nirK* gene. This result suggests that strains EK-V1 and EK-V6 (in NCV and CV) can participate in one of the stages of nitrogen transformation in vinasse, more specifically the transformation of NO^2−^ into NO. Moreover, the strains EK-V1 and EK-V6 are able to produce arginine, essential aminoacid to produce NO^3−^, as well as produce urease enzyme related with urea hydrolysis (Hibbs, Vavrin & Taintor, 1987; Zhou et al., 2019). The heating process to concentrate vinasse reaches 115 °C (Carvalho & Silva, 2010), which drastically decreased *Megasphaera* and *Mitsuokella* genera present in NCV (Fig. 3C), confirming our hypothesis that NCV and CV harbor different bacterial community. In fact, CV harbors mainly *Lactobacillus* genus (Fig. 3C) and the PCoA plots (Fig. 2) are in accordance with this information, showing that there is a possible selection of this genus in CV associated with the evaporation method.

We found for both NCV and CV the dominance of phylum Firmicutes (>90%). Actinobacteria and Bacteroidetes were only present in NCV. Within Firmicutes phylum are bacterial members capable of fermenting various organic substrates and forming spores. Previously, these bacteria group found to be abundant in the ethanol industry process (Costa et al., 2015). Aerobic and anaerobic microbial processes are options for treating vinasse. The vinasses are produced by similar industrial fermentation processes based on the growth of microorganisms on molasses derived from sugarcane. Anaerobic degradation of organic material (biomass), for example, involves decomposition of bacteria under humid condition in the absence of molecular oxygen where different organisms of genera *Actinobacteria*, *Proteobacteria*, *Bacteroidetes* and *Firmicutes* are selected in the different stages of industrial fermentation (Deublein & Steinhauser, 2011; Ray & Bhunia, 2013). Heat-resistant endospores is a specific property of the members of the phylum Firmicutes found in different classes as Bacilli, Clostridia, Erysipelotrichia and Negativicutes, which all encode similar sets of core sporulation proteins (Galperin, 2013). In NCV, the most abundant class was Negativicutes (50%), followed by Bacilli (38%) both belong to Firmicutes while in CV Bacilli corresponded to almost 100% of the total relative abundance.

The application of vinasse on sugarcane fields has negative environmental aspects related to GHG emissions when applied in conjunction with inorganic nitrogen (Lourenço et al., 2018b, 2018d). NCV increases more N_2_O fluxes in the soil than CV (Pitombo et al., 2016). Based on our results, the microbes present in both vinasses, concentrated and non-concentrated, encode genes for denitrification, mainly *nirK*, suggesting the potential capacity of these isolates to contribute to the N_2_O emissions by denitrification when vinasse is applied into soil. Interestingly, three strains from CV (EK-V1 and EK-V3) and NCV (EK-V6) are able to reduce nitrate to nitrite. This step has emerged as an alternative route to the classical enzymatic NO and N_2_O formation.

One issue that should be considered is the substantial amount of vinasse potentially being flushed into soil microbiome in sugarcane cropland. Vinasse may affect the resident microbial activity and relative abundance of specific taxonomic groups in sugarcane-cultivated soils by introducing labile nutrients and exogenous microbes. Traditionally, most studies on biological invasions have focused on invasive plants and animals (Elton, 1958; Mooney & Hobbs, 2000), while only a few considered the effects of invasive microbes, except the study of Lourenço et al. (2018c) with NCV. Previous field and mesocosm experiments showed that vinasse increased abundances of *Bacillaceae*, *Micrococcaceae*, *Hyphomicrobiaceae* and *Nitrospiraceae* families (Pitombo et al., 2016; Navarrete et al., 2015) with functions related to spore-producing microorganisms overrepresented. For instance, Pitombo et al. (2016), Suleiman et al. (2018) and Lourenço et al. (2018d) observed an increase in the abundance of *Lactobacillaceae* in treatments with vinasse, but after 14 days, the relative abundance decreased and was similar to the treatments without vinasse showing that vinasse-exogenous microbes are unable to survive in the soil conditions after certain period. Cassman et al. (2018) identified vinasse-exogenous microbes bacteria that survived the selective bottleneck of the bioethanol production with *Lactobacillu*s (Phylum Firmicutes) being the core genus present in the vinasse input.

The broad functional groups of Gram-positive bacteria dominated in both NCV and CV, over Gram-negative bacteria. Gram-positive bacteria are generally divided into the Actinobacteria and the Firmicutes (Hugget & O’Grady, 2014). This phenomenon is likely due to major differences in the cell envelope between Gram-negatives and Gram-positives. Gram-negative bacteria are the smallest and tend to be more sensitive to disturbances while Gram-positive bacteria are larger, have a thicker cell wall, a negative charge on the outer surface, and tend to resist stress (Mai-Prochnow et al., 2016). Furthermore, the ability of Gram-positive organisms to sporulation may allow them to withstand industrial fermentation process disturbance. The bacterial groups found in this study, including Gram-positive bacteria with low GC as *Bacillus*, *Clostridium*, *Enterococcus*, *Lactobacillus* and *Lactococcus*, which execute the steps of hydrolysis and acidogenesis, were comparable to investigations done in anaerobic digesters (Oude Elferink et al., 1998; Kröber et al., 2009; Sarti et al., 2010).

The isolated strains of this study metabolized different substrates related to different functions. For example, strains EK-V1 and EK-V6 were capable to reduce nitrate to nitrite, hydrolyze arginine and to metabolize urea. Nitrate-nitrite-nitric oxide reductive pathway has emerged as an alternative route to the classical enzymatic NO formation by oxidation of L-arginine with molecular oxygen (Lundberg, Weitzberg & Gladwin, 2008; Bryan & Loscalzo, 2013). This microbial denitrifies present in the soil, possess nitrate reducing mechanisms, maybe responsible for the loss of nitrate (NO_3^−^_) and production of the potent GHG, N_2_O. Urea hydrolysis to NH_4_^+^ and CO_2_ may also take place by microbial uptake (Hoult & McGarity, 1986; Pettit et al., 1976). This could be an indication of microbial activity that makes vinasse an option with available ammonium for soil fertilization since urease activity plays an important role in the regulation of *N* supply to plant after urea fertilization (Fazekašová, 2012). Proteolysis, for instance, is an important process in *N*-cycling because is considered to be a rate-limiting step during *N* mineralization in soils due to the much slower primary phase of protease activities during *N* mineralization compared with amino acids mineralization (Jan et al., 2009). For Beta-galactosidase, probably less bioavailable carbon was present in CV as glycosidase activity facilitates the breakdown of low-molecular-weight carbohydrates and produces the end product-glucose, important in terrestrial C cycling by providing necessary energy for proliferation of microorganisms (Eivazi & Tabatabai, 1990). Glutamine is well known important amino acid in nitrogen metabolism and can be produced by direct fermentation with certain bacteria (Nabe et al., 1980; Nakanishi, 1980). Furthermore, Parnaudeau et al. (2008) reported that sugarcane vinasse contains significant amount of sucrose, oxalate, lactate, malate and pyruvate, which are ready metabolites to be fed to the tricarboxylic acid cycle during the alcoholic fermentation (Bhattacharyya et al., 2012). All these characteristics are related to the bacterial strains isolated from NCV and CV. The phylogenetic analyses based on 16S rRNA gene sequences revealed strains EK-V4 and EK-V5 similar to *Lysinibacillus fusiformis* a species recognized as plant growth promoter (Marasco et al., 2012). The present data indicate that these strains isolated from vinasse could be tested for production of secondary metabolites as well as sugarcane growth-promoting bacteria.

In conclusion, this study shows that process of vinasse concentration reduces the bacterial community diversity. There is the predominance of Firmicutes for both vinasse types capable of fermenting various organic substrates and forming spores. The broad functional groups of Gram-positive bacteria dominated in both NCV and CV, suggest that microbes resist to heating process of vinasse concentration. The strains in both vinasses can metabolize substrates related to the nitrogen cycle by encoding genes for denitrification, mainly *nirK* that might contribute to the N_2_O emissions when vinasse is applied into soil.

## Supplemental Information

10.7717/peerj.6768/supp-1 Supplemental Information 1 Supplemental Figures and Tables. Supplementary Material providing bacterial community composition and Alpha-diversity index (Simpson) of concentrated vinasse (CV) and non-concentrated vinasse (NCV); Chemical composition of concentrated vinasse (CV) and non-cocncentrated (NCV); Primers and PCR conditions of each gene.

## Additional Information and Declarations

### Data Availability

The following information was supplied regarding data availability: The raw sequences are available at the European Nucleotide Archive (ENA) under study accession number PRJEB30243.
