# Supplementary material for: Bacterial community composition and diversity of two different forms of an organic residue of bioenergy crop
Source: PeerJ. 2019 Apr 18;7:e6768. doi: 10.7717/peerj.6768 (PMC6475576; doi:10.7717/peerj.6768)
Supplement: Supplemental Information 1 — Supplementary Material providing bacterial community composition and Alpha-diversity index (Simpson) of concentrated vinasse (CV) and non-concentrated vinasse (NCV); Chemical composition of concentrated vinasse (CV) and non-cocncentrated (NCV); Primers and PCR conditions of each gene. [file peerj-07-6768-s001.docx]

Supporting information


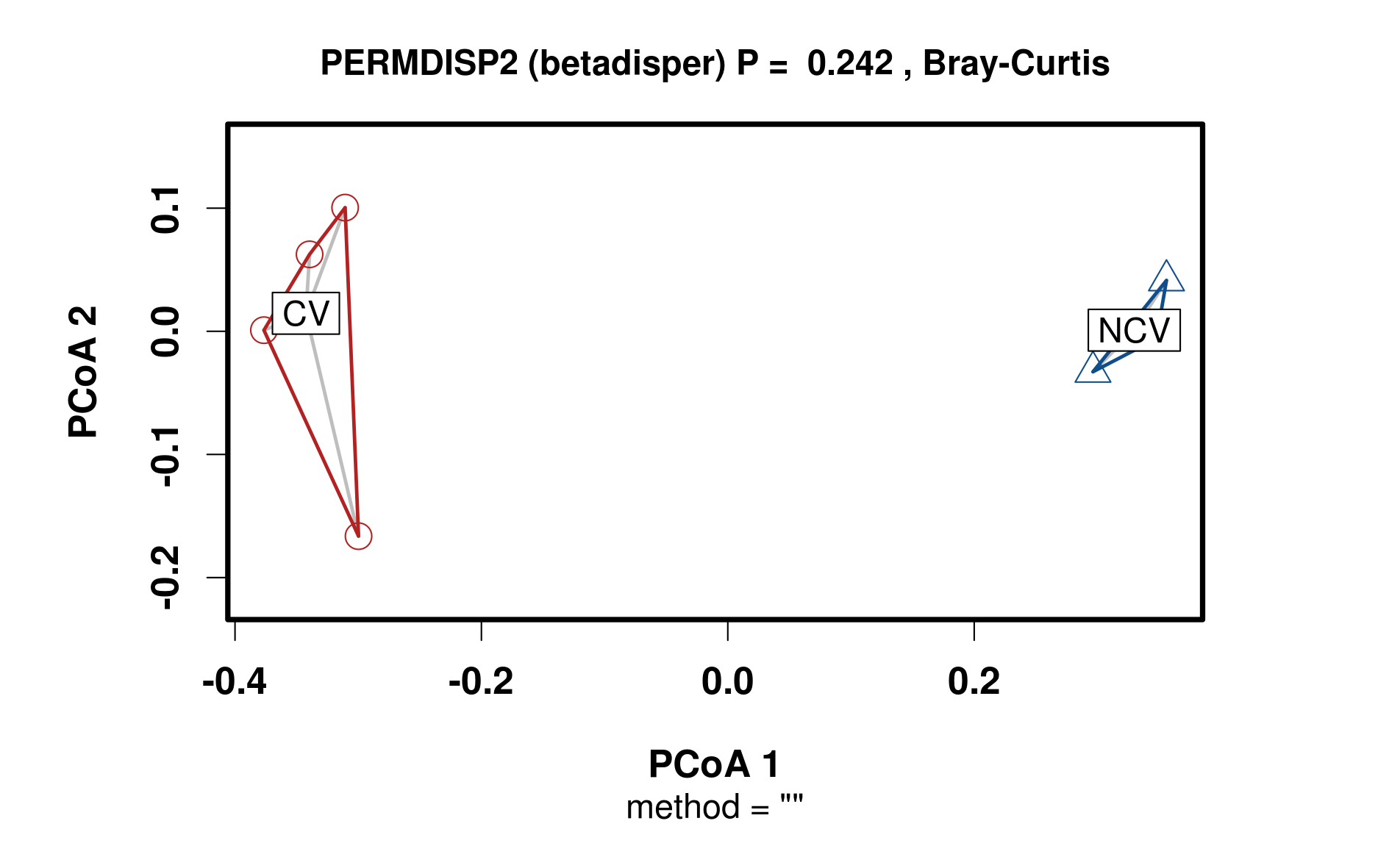


Fig. S1. Permutational multivariate dispersion (PERMDISP) test of bacterial community composition of concentrated vinasse (C) and non-concentrated vinasse (N)


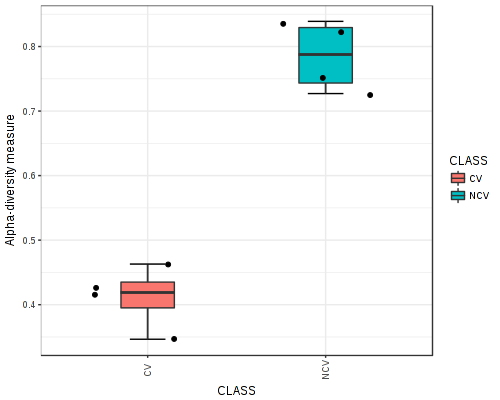


Fig. S2. Alpha-diversity index (Simpson) of concentrated vinasse (CV) and non-concentrated vinasse (NCV).

Table S1. Chemical characteristics of the concentrated vinasse (CV) and non-cocncentrated (NCV).

|  | pH | C org^b^ | N tot ^c^ | NH_4_^+^-N ^d^ | NO_3_^-^N ^e^ | P | K | C/N |
| --- | --- | --- | --- | --- | --- | --- | --- | --- |
|  |  | g L^-1^ | g L^-1^ | mg L^-1^ | m g L^-1^ | g kg^-1^ | g kg^-1^ |  |
| CV | 4.8 | 28.8 | 0.51 | 45.7 | 8.8 | 0.11 | 3.5 | 57/1 |
| NCV | 3.9 | 31.4 | 0.89 | 41.6 | 4.1 | 0.23 | 4.7 | 35/1 |

^b^ C org: Total organic carbon;

^c^ N tot:Total organic nitrogen;

^d^NH_4_^+^-N: ammonium;

^e^ NO_3_^-^-N: nitrate.

Table S2. Primers and PCR conditions of each gene.

| Gene | Primer | Reference |
| --- | --- | --- |
| *narG* | narG1960m2F | Bru et al., 2007 |
|  | narG2050m2R |  |
| *nirK* | nirK 1F | Braker et al., 1998 |
|  | nirK 5R |  |
| *nirS* | nirScd3af | Throback et al., 2004 |
|  | nirSR3cd |  |
| nosZ | nosZ2F | Henry et al., 2006 |
|  | nosZ2R |  |
| *amoA* | AmoA-1F | Rotthauwe et al., 1997 |
|  | AmoA-2R-TC |  |
| *nifH* | PolF | Poly et al. (2001) |
|  | PolR |  |
